# Map-Based Cloning and Characterization of *Br-dyp1*, a Gene Conferring Dark Yellow Petal Color Trait in Chinese Cabbage (*Brassica rapa* L. ssp. *pekinensis*)

**DOI:** 10.3389/fpls.2022.841328

**Published:** 2022-02-17

**Authors:** Shuangjuan Yang, Honglei Liu, Yanyan Zhao, Henan Su, Xiaochun Wei, Zhiyong Wang, Xiaobin Zhao, Xiao-Wei Zhang, Yuxiang Yuan

**Affiliations:** ^1^Institute of Horticulture, Henan Academy of Agricultural Sciences, Zhengzhou, China; ^2^School of Agricultural Sciences, Zhengzhou University, Zhengzhou, China

**Keywords:** *Brassica rapa*, flower color, dark yellow petal color, fine-mapping, breeding

## Abstract

Flower color is an important trait in *Brassica* species. However, genes responsible for the dark yellow flower trait in Chinese cabbage have not been reported. In this study, we identified a dark-yellow-flowered Chinese cabbage line SD369. Genetic analysis indicated that the dark yellow flower trait in SD369 was controlled by a single recessive locus, *Br-dyp1* (*dark yellow petal color* 1 in *Brassica rapa*). Using bulked segregant RNA sequencing and kompetitive allele-specific PCR assays, *Br-dyp1* was fine-mapped to an interval of 53.6 kb on chromosome A09. Functional annotation analysis, expression analysis, and sequence variation analysis revealed that *Bra037130* (*BraA09.ZEP*), which encodes a zeaxanthin epoxidase, was the most likely candidate gene for *Br-dyp1*. Carotenoid profile analysis suggested that *Bra037130* (*BraA09.ZEP*) might participate in the epoxidation from zeaxanthin to violaxanthin. The 679 bp insertion in dark yellow petal caused premature stop codon, thus caused the loss-of-function of the enzyme zeaxanthin epoxidase (ZEP), which disturbed the carotenoid metabolism, and caused the increased accumulation of total carotenoid, and finally converted the flower color from yellow to dark yellow. Comparative transcriptome analysis also showed that the “carotenoid biosynthesis” pathway was significantly enriched, and genes involved in carotenoid degradation and abscisic acid biosynthesis and metabolism were significantly downregulated. Furthermore, we developed and validated the functional marker Br-dyp1-InDel for *Br-dyp1*. Overall, these results provide insight into the molecular basis of carotenoid-based flower coloration in *B. rapa* and reveal valuable information for marker-assisted selection breeding in Chinese cabbage.

## Introduction

Flower color is one of the most important traits in *Brassica* species and is particularly useful for ornamental and landscaping purposes (Zhang et al., 2018a; Liu et al., 2020b). In breeding, flower color can be used to evaluate variety purity in hybrid production (Zhang et al., 2018b; Yang et al., 2021). *Brassica* flowers are usually yellow, but can also be white, milky white, orange, or dark yellow (Zhang et al., 2018a). The gene *carotenoid cleavage dioxygenase 4* (*CCD4*) underlies the white flower trait in *Brassica napus* and *Brassica oleracea* (Zhang et al., 2015; Han et al., 2019). In *Brassica rapa*, the orange flower color as well as the orange coloration of the inner leaves is controlled by the *carotenoid isomerase* (*BrCRTISO*) gene (Su et al., 2014). Our previous study showed that the *BrWF3* gene, which encodes a diacylglycerol acyltransferase and is homologous to *PES2* in *Arabidopsis*, controls the white flower trait in Chinese cabbage (*B. rapa* L. ssp. *pekinensis*; Yang et al., 2021). However, the gene underlying the dark yellow flower trait in Chinese cabbage or in *B. rapa* species has not been reported.

Carotenoids are a group of more than 700 lipid-soluble pigments synthesized in plastids (Gonzalez-Jorge et al., 2016; Li et al., 2016). In chloroplasts, carotenoids are essential structural and functional components of the antenna complex of the photosynthesis system, with roles in light harvesting, non-photochemical quenching, and limiting membrane damage by reactive oxygen species and singlet oxygen species (Walter and Strack, 2011; Zhang et al., 2015; Gonzalez-Jorge et al., 2016). Carotenoids in chromoplasts endow flowers and fruits with different colors, such as orange, yellow, and red, which attract animals for pollination and seed dispersal (Kevan and Baker, 1983). Carotenoids are also biosynthetic precursors for the synthesis of the plant hormones abscisic acid (ABA) and strigolactone (Frey et al., 2006; Alder et al., 2012).

The enzyme zeaxanthin epoxidase (ZEP) plays a critical role in carotenoid biosynthesis, which is responsible for the epoxidation of zeaxanthin to yield antheraxanthin and subsequently violaxanthin. Violaxanthin can be deepoxidated to antheraxanthin and then zeaxanthin by the enzyme violaxanthin de-epoxidase. This reversible epoxidation/deepoxidation is referred to as the xanthophyll cycle, in which deepoxidation to zeaxanthin is favored under high-light conditions while epoxidation to violaxanthin predominates in moderate-light conditions. The rapid formation of zeaxanthin *via* the xanthophyll cycle is indispensable for the dissipation of excess energy by non-photochemical quenching, while violaxanthin is a precursor for ABA biosynthesis; thus, xanthophyll cycle is one of the critical processes contributing to plant fitness and stress tolerance (Alboresi et al., 2011; Gao et al., 2013; Gonzalez-Jorge et al., 2016; Lou et al., 2017). Many studies have showed that ZEP proteins are involved in ABA biosynthesis, *ZEP* mutants display low ABA levels and almost no ABA upregulation under drought stress, while the overexpression of *ZEP* enhances tolerance to osmotic stress, which suggests that the ZEP enzyme plays a critical role in the ABA-mediated stress response (Marin et al., 1996; Agrawal et al., 2001; Xiong et al., 2002; Park et al., 2008). However, the impact of ZEP on carotenoid pigmentation in the flowers of Chinese cabbage has not been investigated.

In this study, we conducted positional cloning of the dark yellow petal color gene (*Br-dyp1*) in Chinese cabbage by using F_2_ populations, which derived from the dark-yellow-flowered inbred line “SD369” and the yellow-flowered DH line “R16-11.” Furthermore, we conducted carotenoid profile analysis and comparative transcriptome analysis to figure out the mechanisms underlying the dark yellow flower color pigmentation. In addition, we developed and validated a functional marker. This work will promote marker-assisted selection breeding and the exploration of molecular mechanisms that regulate flower color variation in Chinese cabbage or in *B. rapa*.

## Materials and Methods

### Plant Materials

The yellow-petaled double haploid (DH) line R16-11 (P_1_) and the dark-yellow-petaled inbred Chinese cabbage line SD369 (P_2_) were used as parents to generate F_1_, F_2_, BC_1_P_1_, and BC_1_P_2_ populations for the inheritance and mapping studies. The BC_1_P_1_ and BC_1_P_2_ populations were created by backcrossing F_1_ plants with R16-11 or SD369, respectively. The petal color trait was investigated visually at the flowering stage. Statistical analyses of the segregation ratios of the F_2_ and BC_1_P_1_ populations were carried out through chi-square test (*χ*^2^). Additionally, eight yellow-petaled materials, eight dark-yellow-petaled materials, eight orange-petaled materials, and eight white-petaled materials were used to analyze mutations in the candidate gene (Supplementary Table 1). All the materials used in this study were provided by the Institute of Horticulture, Henan Academy of Agricultural Sciences.

### Carotenoids Identification and Quantification

Carotenoids composition was measured by MetWare^1^ based on the AB Sciex QTRAP 6500 LC–MS/MS platform. Petals from 10 dark-yellow-petaled F_2_ plants were combined to form one replicate (referred to as the DY-bulk), and petals from 10 yellow-petaled F_2_ plants were included in the Y-bulk. In total, three replicates were assessed. Fresh petals were freeze-dried, ground into powder (30 Hz, 1.5 min), and stored at −80°C until needed. For each sample, 50 mg powder was weighted and extracted with 0.5 ml of a mixed solution of n-hexane: acetone: ethanol (1:1:1, v/v/v) with 0.01% BHT (g/ml), and 10 μl of (13C10)-β-carotene solution (20 μg/ml) were added into the extract as internal standards for quantification. The extract was vortexed for 20 min at room temperature. The supernatants were collected after centrifugation at 12000 r/min for 5 min at 4°C. The residue was re-extracted by repeating the above steps again under the same conditions. Saturated sodium chloride solution (0.5 ml) was added to the supernatant, after which the mixture was vortexed, and the upper layer was collected. This step was repeated two times more. Then, the supernatant was evaporated to dryness and dissolved in 0.5 ml of MTBE, then 0.5 ml 10% KOH-MeOH was added, the mixture was vortexed again, and the reaction was allowed to take place at room temperature overnight. After the reaction, 1 ml of saturated sodium chloride solution and 0.5 ml of MTBE were added, followed by vortexing, and the upper layer was collected. This step was repeated two times, and the supernatant was evaporated to dryness and reconstituted in 100 μl of mixed solution of MeOH/MTBE (1:1, v/v). The solution was filtered through a 0.22 μm membrane filter for further LC–MS/MS analysis.

The sample extracts were analyzed using a UPLC-APCI-MS/MS system (UPLC, ExionLC^™^ AD; MS, Applied Biosystems 6,500 Triple Quadrupole), which was performed as described in previous studies (Liu et al., 2020a; Wang et al., 2020; Zhou et al., 2020; Yang et al., 2021). The integrated peak area of each carotenoid detected in the samples was substituted into the linear equations of standard curves for content calculation; finally, the absolute content data for the carotenoids in the actual samples were obtained (Supplementary Table 2). The specific procedure for calculation of the carotenoid content was performed as Yang et al. (2021).

### Bulked Segregant RNA Sequencing and Analysis

The DY-bulk and Y-bulk each with three replicates used for carotenoid analysis were also used for RNA sequencing. Six cDNA were constructed and sequenced at BioMarker Tech Co., Ltd. (Beijing, China). The Illumina HiSeq X 10 platform was used to generate 150-base paired-end reads for each library. To preliminarily map the candidate gene, the clean reads from the three DY-bulk replicates were merged to form a single read file, and another merged file was obtained from the three Y-bulk replicates. Then, the merged read files were aligned to the *B. rapa* reference genome (V1.5) using BWA software (Li and Durbin, 2010). The single-nucleotide polymorphism (SNP) and insertion/deletion (InDel) variants were called using SAMtools software (Li et al., 2009). The SNP index was calculated for all genomic positions in the DY-bulk and Y-bulk and the Δ(SNP index) was calculated by subtracting the SNP index of the Y-bulk from that of the DY-bulk, which was performed as previously described (Abe et al., 2012; Takagi et al., 2013; Yang et al., 2021). The candidate region of *Br-dyp1* was identified by sliding window analysis with a 1-Mb width and a 50-kb increment at the 95% confidence level according to (Yang et al., 2021).

To identify differentially expressed genes (DEGs) between the DY-bulk and Y-bulk, the clean reads of each library were aligned to the *B. rapa* V1.5 reference genome using HISAT2 software with the default parameters (Kim et al., 2015). Then, the fragments per kilobase of transcript per million mapped reads value of each gene was calculated to estimate gene expression levels. DEGs were identified using the DESeq2 package (v1.6.3; Love et al., 2014). Genes with false discovery rate (FDR) ≤ 0.05 and |log2 (fold change) | ≥ 1 were recognized as DEGs. Gene Ontology (GO) enrichment analysis was carried out using the topGO package (v2.18.0; Alexa et al., 2010). Kyoto Encyclopedia of Genes and Genomes (KEGG) pathway enrichment analysis was implemented using KOBAS (v2.0) software (Mao et al., 2005; Wu et al., 2006).

### Kompetitive Allele-Specific PCR Marker and Linkage Map Development

To validate the results of Bulked Segregant RNA Sequencing (BSR-Seq) and map the *Br-dyp1* gene, we selected SNPs showing polymorphism between the two bulks and nearing the candidate region for Kompetitive Allele-Specific PCR (KASP) marker development. The detailed procedures for KASP marker development and KASP assays were performed as described by (Yang et al., 2020). The developed KASP markers were first screened between R16-11 and SD369. Then, polymorphic KASP markers (Supplementary Table 3) were employed to genotype the F_2_ population containing 94 individuals. The genetic linkage map was constructed using JoinMap 4.0 software (Van Ooijen, 2006). Recombination values were converted into genetic map distances (cM) following the Kosambi mapping function (Kosambi, 1943).

For the fine-mapping of the candidate gene, approximately 3,500 F_2_ seeds were planted, and 743 individuals with a dark yellow petal phenotype were used for screening more recombinants.

### Cloning and Sequence Analysis of the Candidate Genes

We designed primer pairs according to the *B. rapa* reference genome to clone the DNA and cDNA sequence of the candidate gene. Phanta Flash Master Mix (Vazyme Biotech Co., Ltd., Nanjing, China) was deployed to amplify the candidate gene. The PCR products were sequenced by Sunya Biotech Co., Ltd. (Zhengzhou, China), and sequence alignments were performed using DNAMAN software. The coding sequences (CDSs) of candidate gene from R16-11 and SD369 were submitted to GenBank under the accession numbers OL436220 (R16-11) and OL436221 (SD369).

### Quantitative Real-Time PCR

Total RNA was extracted from tissues of roots, stems, leaves, sepals, petals, stamens, and pistils from R16-11 and SD369. *BrGAPDH* was used as an internal control (Qi et al., 2010; Su et al., 2014). Quantitative Real-Time PCR (qRT-PCR) was performed with 2 × TB Green Premix Ex Taq II (TaKaRa, Japan) on a Roche LightCycler 480-II System (Roche Applied Sciences, Beijing, China). Relative expression levels were calculated using the 2^−ΔΔCt^ method (Livak and Schmittgen, 2001). The results from three biological replicates are shown.

## Results

### The Dark Yellow Petal Color Trait of SD369 Is Controlled by a Single Recessive Gene

**Figure 1:**
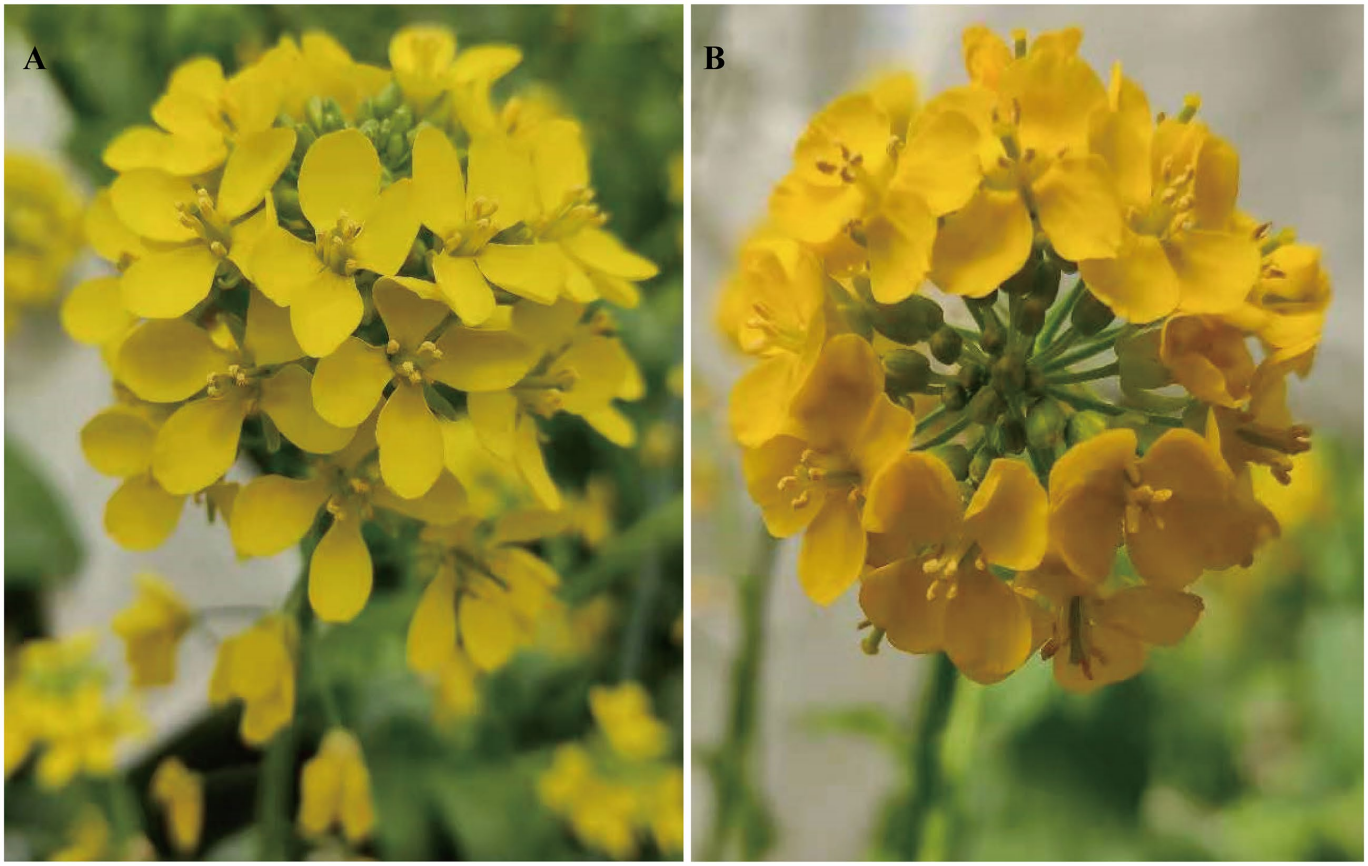
Phenotypic characterization of flower color in the two parent lines (R16-11 and SD369). The flower color of R16-11 **(A)** is yellow at the flowering stage, while dark yellow for SD369 **(B)**.

The phenotypic analysis showed significant differences in petal color between the two parental lines (Figure 1). In R16-11 (P_1_), the petals showed stable yellow coloration at the flowering stage, whereas those of SD369 (P_2_) exhibited dark yellow coloration (Figures 1A,B). All 15 individuals in the F_1_ population showed a yellow petal phenotype, as did R16-11. Phenotypic segregation was observed in the F_2_ and BC_1_P_2_ populations, and the petals of the plants in these two populations exhibited two kinds of colorations, yellow or dark yellow, corresponding to the coloration of either R16-11 or SD369, respectively. In a small F_2_ population, 176 plants exhibited yellow petals, and 54 showed dark yellow petals, corresponding to a segregation ratio of 3:1 by the chi-square test (Table 1). In a larger F_2_ population, the segregation ratio was also 3:1 (2,371 yellow:743 dark yellow, *χ*^2^ = 2.16). The numbers of individuals with yellow and dark yellow petals in the BC_1_P_2_ population were 125 and 115, respectively, corresponding to a ratio of 1:1 according to the chi-square test (*χ*^2^ = 0.42 < $\chi_{0.05}^{2}$ = 3.84, *p* < 0.05). Furthermore, all 200 BC_1_P_1_ plants showed yellow petals. All of these results demonstrate that the dark yellow petal color trait of SD369 is controlled by a monogenic recessive gene (Table 1). We named this locus *Br-dyp1* (*dark yellow petal color 1 in B. rapa*).

**Table 1:** Genetic analysis of the petal trait in parents and in crosses between R16-11 and SD369.

Generations	Total	Yellow	Dark yellow	Expected ratio	*χ* ^2^	$\chi_{0.05}^{2}$
P_1_ (R16-11)	10	10	0	–	–	–
P_2_ (SD369)	10	0	10	–	–	–
F_1_	15	15	0	–	–	–
F_2_-small	230	176	54	3:1	0.28	3.84
F_2_-large	3,114	2,371	743	3:1	2.16	3.84
BC_1_P_1_ (F_1_ × R16-11)	200	200	0	–	–	–
BC_1_P_2_ (F_1_ × SD369)	240	125	115	1:1	0.42	3.84

### The Carotenoid Profile Is Altered in Dark Yellow Petals

**Figure 2:**
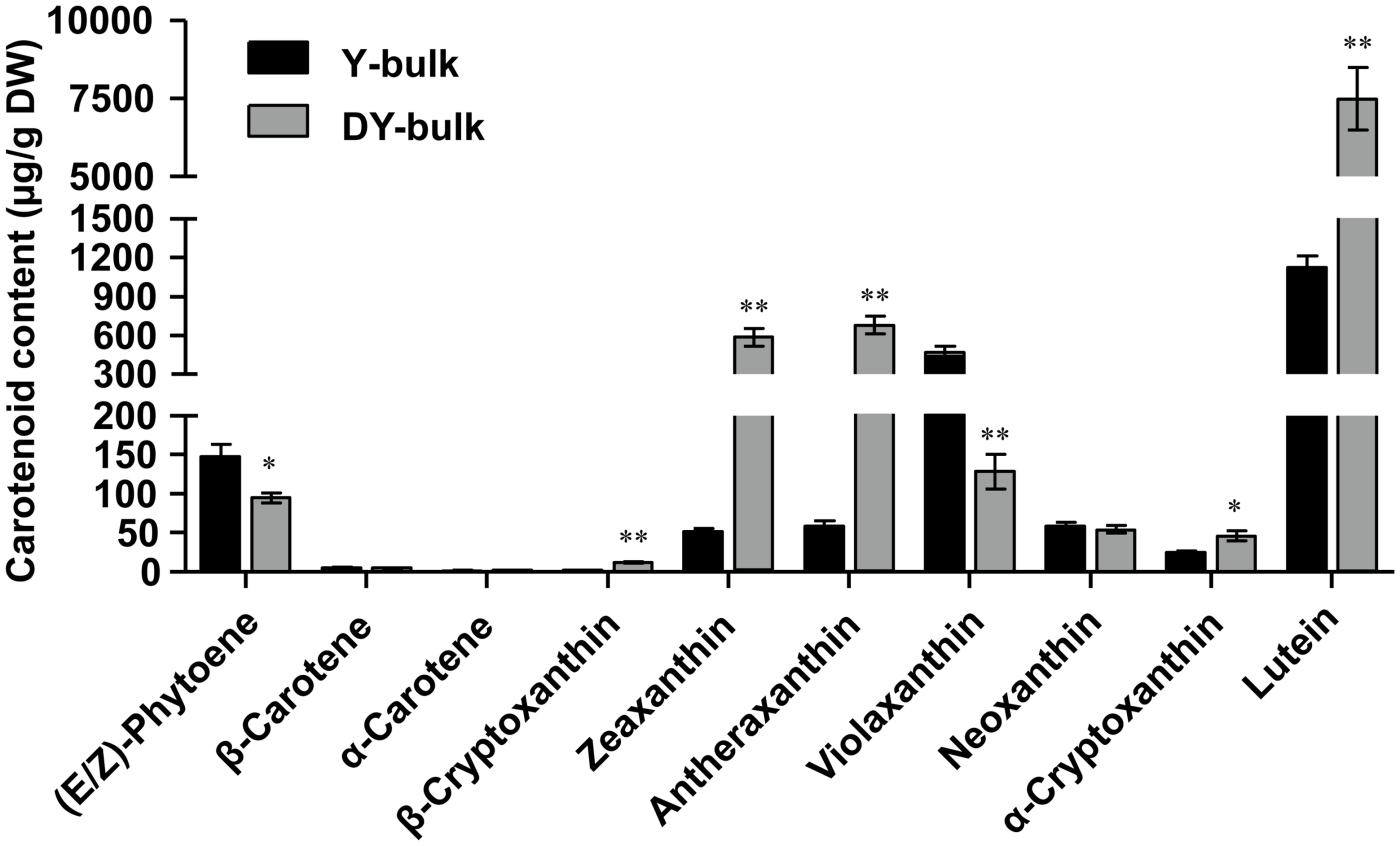
Carotenoid composition in petals from Y-bulk and DY-bulk. Carotenoids extracts from yellow and dark yellow petals were subjected to UPLC-APCI-MS/MS system under saponification treatment. Error bars indicate SE (*n* = 3). Value of ^*^p < 0.05 (Student’s *t*-test). Value of ^**^p < 0.01 (Student’s *t*-test).

The composition and content of carotenoids in petals of the Y-bulk and DY-bulk were determined using UPLC-APCI-MS/MS system under saponification treatment. The results showed that the carotenoid profiles of the DY-bulk were quite different from those of the Y-bulk. Ten carotenoids composition were identified in Y-bulk and DY-bulk, including three carotenes and seven xanthophylls (Figure 2; Supplementary Table 4). The total content of carotenes was slightly lower in the DY-bulk than in the Y-bulk (Supplementary Table 4). The total content of xanthophylls accounted for approximately 92.0% or 98.9% of the total carotenoids in the Y-bulk and DY-bulk, respectively, and the contents of almost all xanthophylls were higher in the DY-bulk than in the Y-bulk. For example, the amounts of lutein, zeaxanthin, and antheraxanthin in the DY-bulk increased about 6.7-fold, 11.2-fold, and 11.5-fold than that in Y-bulk. However, the amount of violaxanthin decreased about 3.6-fold in DY-bulk (Figure 2; Supplementary Table 4). The total content of carotenoids showed a remarkable 4.7-fold increase in dark yellow petals relative to that in the yellow petals (Supplementary Table 4). Taken together, these findings suggested that the dark yellow petal phenotype in SD369 resulted from increased contents of carotenoids, particularly lutein, zeaxanthin, and antheraxanthin.

### Fine-Mapping of the *Br-dyp1* Gene

*Br-dyp1* was preliminarily mapped using BSR-seq. A total of 47,206,290, 38,634,054, and 54,612,412 clean reads were obtained for the three Y-bulks, while 47,676,208, 51,940,890, and 49,967,262 clean reads were obtained for the three DY-bulks (Supplementary Table 5). Reads from the three Y-bulks were merged as the Y-pool, and reads from the three DY-bulks were merged to form the DY-pool. The Y-pool and DY-pool clean reads were aligned to the *B. rapa* V1.5 genome, and a total of 348,456 SNPs and 29,092 InDels were identified between these two pools. The Δ(SNP index) of each position was calculated for sliding window analysis. According to the null hypothesis, a 2.7 Mb region from 3.8 to 6.5 Mb on chromosome A09 exhibiting significant linkage disequilibrium was identified as the candidate region for the dark yellow petal trait at a 99% confidence level (Supplementary Figure 1; Supplementary Table 6), which was consistent with the genetic analysis showing the dark yellow petal color trait was controlled by a single recessive nuclear gene.

**Figure 3:**
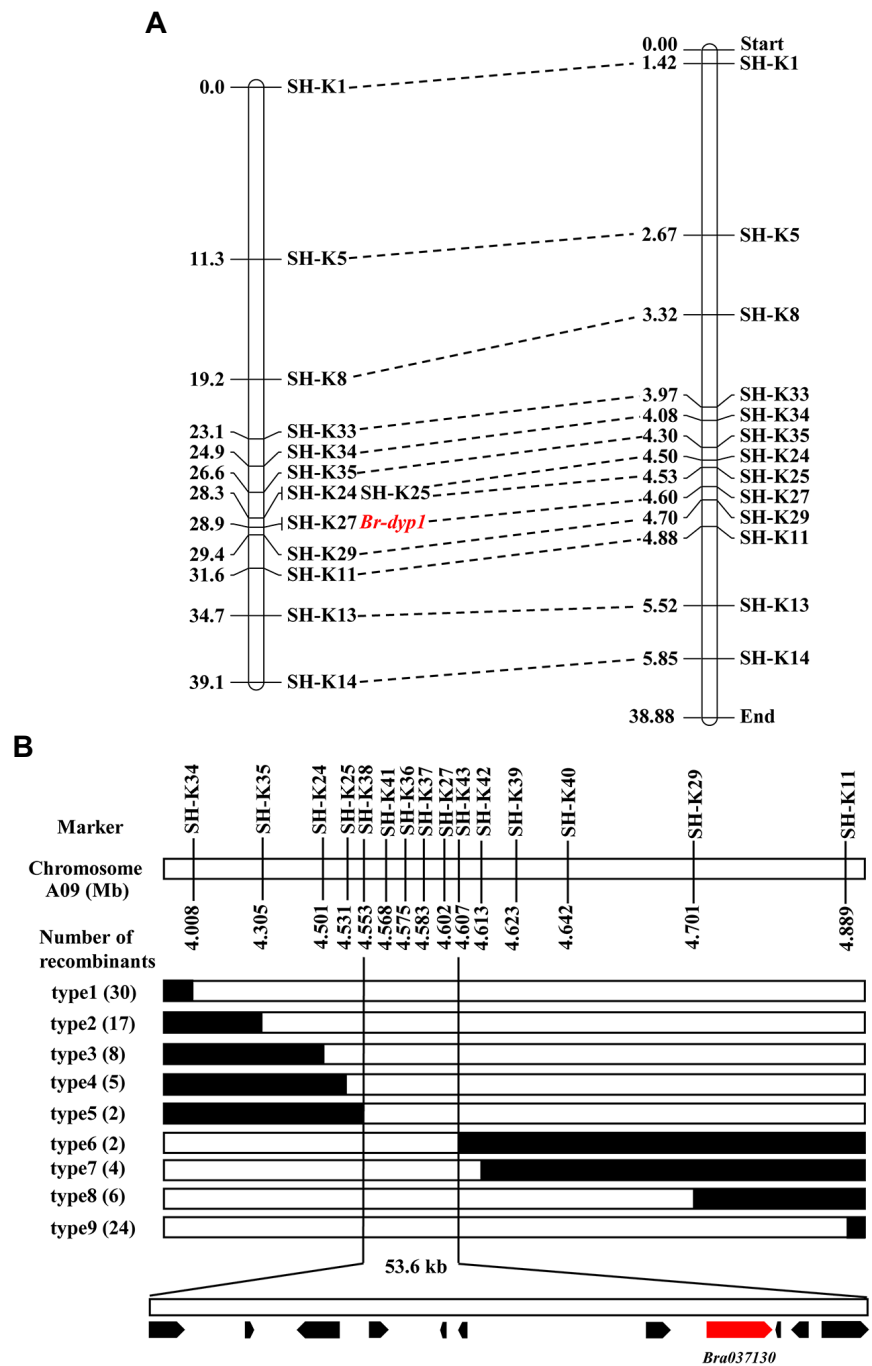
Initial and fine-mapping of the *Br-dyp1* gene in Chinese cabbage. **(A)** Initial mapping of *Br-dyp1*. Genetic map of *Br-dyp1* is on the left, with cM as the unit. The corresponding physical map (right, unit: Mb) are also shown. **(B)** Fine-mapping of *Br-dyp1*. The *Br-dyp1* gene was delimited to an interval between SH-K38 and SH-K43 on chromosome A09, with an estimated physical length of 53.6 kb, and 11 genes were annotated in this region based on the reference genome sequence. The genetic structure of each recombinant type is depicted as white for homozygous dark yellow petal color phenotype, black for heterozygous alleles, respectively. The number of each recombinant type is indicated in the brackets.

To validate the BSR-Seq results, 35 KASP markers in the candidate region were developed and used to screen the two parents. The results showed that 13 KASP markers (Supplementary Table 3) exhibited good polymorphism. These 13 markers were further genotyped in 94 F_2_ plants for linkage analysis (Supplementary Table 7). The results showed that the marker SH-K27 co-segregated with the *Br-dyp1* gene in the preliminary mapping population (Figure 3A). There was one recombinant individual between *Br-dyp1* and SH-K25 and SH-K29. The genetic distances between the *Br-dyp1* locus and SH-K25 and SH-K29 were 0.6 and 0.5 cM, respectively (Figure 3A). The order of the markers in the genetic map is consistent with that in the physical map (Figure 3A).

To fine-map the *Br-dyp1* locus, we screened 743 dark-yellow-petaled F_2_ plants using the flanking markers SH-K34 and SH-K11 and identified 54 recombinants. All the 54 recombinants were further genotyped using SH-K35, SH-K24, SH-K25, SH-K27, and SH-K29, based on which 11 recombinants (type 4 and type 8) were identified (Figure 3B). Then, eight new markers were developed (Supplementary Table 3) and were further used to screen all the 11 recombinants using the KASP assay. The results delimited the *Br-dyp1* gene to a 53.6 kb interval between markers SH-K38 and SH-K43, each with two recombinants (type 5 and type 6; Figure 3B). Four markers, namely, SH-K41, SH-K36, SH-K37, and SH-K27, co-segregated with the *Br-dyp1* gene in the fine-mapping population (Figure 3B).

### Candidate Gene Analysis

DNA sequences in the fine-mapping interval (53.6 kb) of *Br-dyp1* were analyzed according to the *B. rapa* reference genome. Totally, 11 annotated or predicted genes were identified in the mapping region (Table 2). Among them, *Bra037130* (*BraA09.ZEP*), a homolog of *ZEP* in *Arabidopsis*, could be the candidate gene (Table 2). *ZEP* encodes a zeaxanthin epoxidase that catalyzes the conversion of zeaxanthin to antheraxanthin and violaxanthin in the carotenoid biosynthesis pathway (Liu et al., 2020b).

**Table 2:** Annotated genes in the candidate interval of the *Br-dyp1* locus.

Gene name	Gene position on A09	Arabidopsis homolog	Gene function
*Bra037123*	4,553,464–4,555,964	*AT3G46730*	NB-ARC domain-containing disease resistance protein
*Bra037124*	4,560,614–4,561,153	*AT5G67190*	Encodes a member of the DREB subfamily A-5 of ERF/AP2 transcription factor family; DEAR2
*Bra037125*	4,564,557–4,567,632	*AT5G67170*	SEC-C motif-containing protein
*Bra037126*	4,569,807–4,571,165	*AT5G67160*	Encodes a member of the BAHD acyltransferase superfamily; EPS1
*Bra037127*	4,575,174–4,575,557	*AT5G67070*	Rapid Alkalinization Factor; RALF34
*Bra037128*	4,576,524–4,577,141	*AT5G67060*	Encodes a bHLH transcription factor; HEC1
*Bra037129*	4,590,446–4,592,182	*AT5G67050*	alpha/beta-Hydrolases superfamily protein
*Bra037130*	4,594,979–4,599,768	*AT5G67030*	Zeaxanthin epoxidase; ZEP
*Bra037131*	4,600,167–4,600,430	*AT5G66985*	Hypothetical protein; HUP44
*Bra037132*	4,601,342–4,602,476	*AT5G66980*	AP2/B3-like transcriptional factor family protein
*Bra037133*	4,603,501–4,606,981	*AT5G66960*	Prolyl oligopeptidase family protein

**Figure 4:**
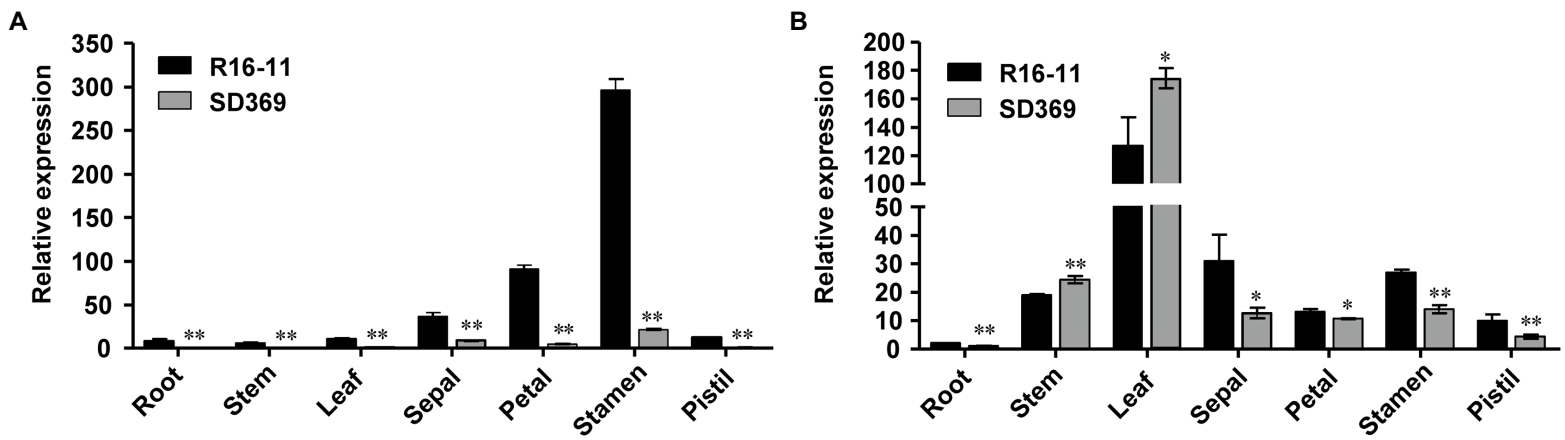
Gene expression data analysis. Quantitative RT-PCR of *Br-dyp1* (*Bra037130*; **A**) and *BraA07.ZEP* (*Bra012127*; **B**) in different tissues of the two parents. The *BrGAPDH* were used as an internal control. Error bars indicate SE (*n* = 3). Value of ^*^p < 0.05 (Student’s *t*-test). Value of ^**^p < 0.01 (Student’s *t*-test).

Next, we examined the expression of the candidate gene *Bra037130* (*BraA09.ZEP*) in different tissues of the two parent lines. qRT-PCR analysis using primer pairs Br-dyp1-qF1 and Br-dyp1-qR1 (Supplementary Table 8) revealed that the expression pattern of *Bra037130* was significantly different between the parental lines. In any of the seven tissues that we examined, the expression of *Bra037130* was much lower in the dark-yellow-petaled parent SD369 than in the yellow-petaled parent R16-11 (Figure 4A). The highest levels of *Bra037130* (*BraA09.ZEP*) were present in the stamens and petals in both parental lines (Figure 4A).

**Figure 5:**
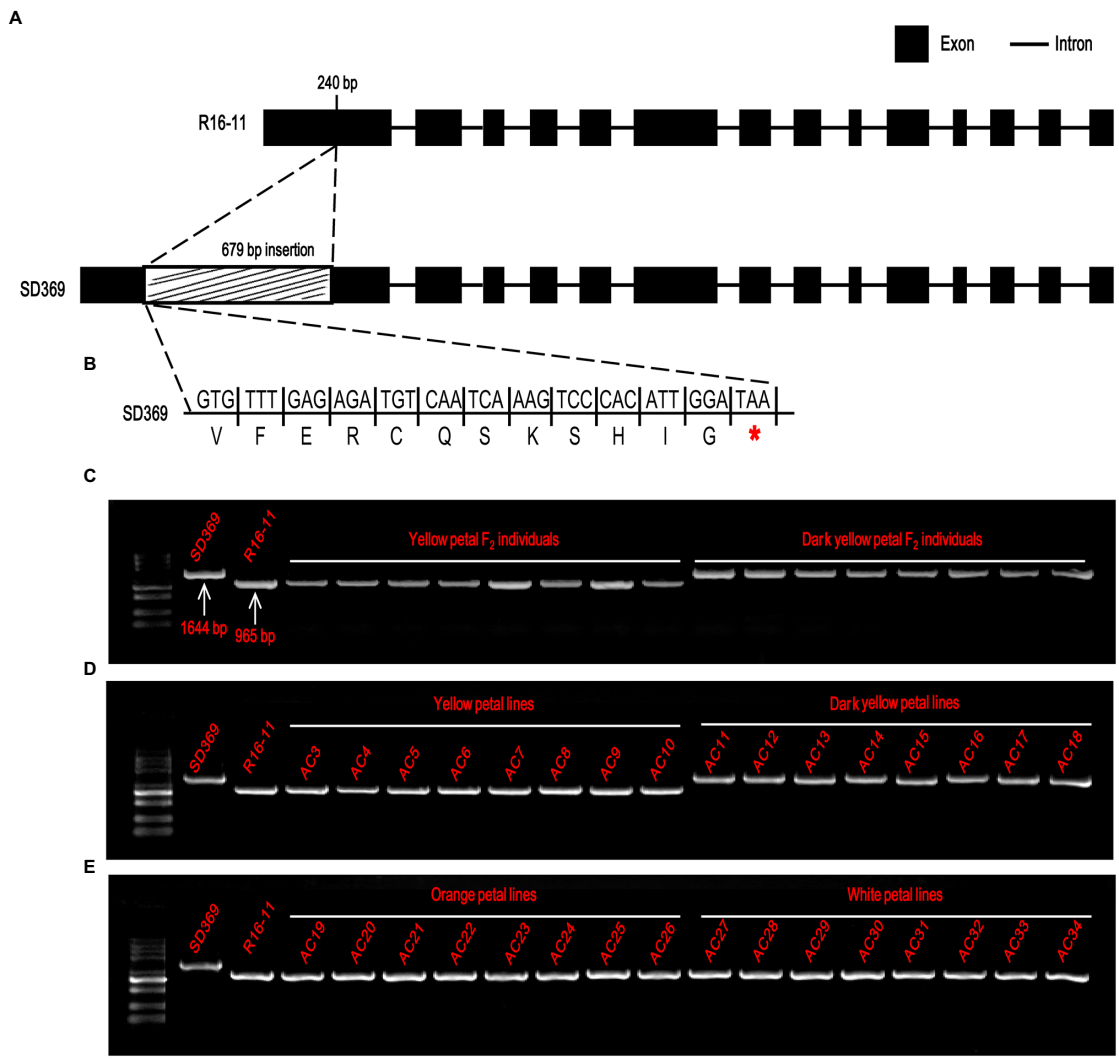
Candidate gene analysis of *Br-dyp1*. **(A)** *Br-dyp1* includes 14 exons and 13 introns in the two parents. **(B)** A 679-bp insertion in dark-yellow-flowered SD369 caused a premature stop codon. **(C–E)** Validation of the functional marker Br-dyp1-InDel in F_2_ individuals **(C)**, in 8 yellow and 8 dark-yellow-flowered materials **(D)**, and in eight orange and eight white flowered materials **(E)**.

To characterize the sequence of the candidate genes in the parental lines, the primer pair Br-dyp1-F and Br-dyp1-R2 (Supplementary Table 8) were designed. Sequence analysis indicated that the candidate gene of R16-11 was 3,020 bp in length and contained 14 exons and 13 introns (Figure 5A; Supplementary Figure 2). The CDS of the candidate gene in R16-11 was 1965 bp in length (Supplementary Figure 2). Sequence alignment showed that there were 43 SNP variations and 11 InDel variations between the genomic sequences of R16-11 and SD369 (Supplementary Table 9; Supplementary Figure 3). Among these variations, the most significant sequence variation was a 679 bp insertion located at 240 bp of the gDNA, within the first exon in SD369 (Figure 5A; Supplementary Table 10). The 679 bp insertion caused a premature stop codon at the 93 a.a position (Figure 5B). Amino acid sequence alignment indicated that the deduced amino acid sequence of *Bra037130* (*BraA09.ZEP*) in R16-11 was highly identical to the ZEP protein sequence in *Arabidopsis* (Supplementary Figure 4), and it contained four conserved motifs: two lipocalin conserved motifs (145–162 a.a and 264–283 a.a), a long monooxygenase domain (223–429 a.a), and a Forkhead-associated domain (579–625 a.a; Supplementary Figure 4). The 679 bp insertion in *BraA09.ZEP* caused the loss of the four conserved domains and ultimately resulted in the loss of function of the ZEP protein in dark yellow petals.

Based on the 679 bp insertion in dark-yellow-petaled parent SD369, a functional marker Br-dyp1-InDel (primers pair Br-dyp1-ful-F and Br-dyp1-sp-R1; Supplementary Table 8), which could amplify a 1,644-bp and 965-bp product from line SD369 and R16-11, respectively, were developed and were assayed in different materials. The results showed that Br-dyp1-InDel co-segregated with the petal color phenotype in the F_2_ population (Figure 5C). Furthermore, eight yellow-petaled materials, eight dark yellow-petaled materials, eight orange-petaled materials, and eight white-petaled materials were genotyped using Br-dyp1-InDel. As expected, all the eight dark yellow-petaled materials showed the same genotype as SD369, and all eight yellow-petaled materials showed the same genotype as R16-11 (Figure 5D). Interestingly, the eight orange-petaled and eight white-petaled materials also exhibited the same genotype as R16-11 (Figure 5E), which implied that the genes controlling the orange and white petal color trait were different from *BraA09.ZEP* and the 679 bp insertion only existed in dark-yellow-petaled materials. Overall, these findings suggest that the *Bra037130* (*BraA09.ZEP*) gene is the most promising candidate gene for the dark yellow petal color gene *Br-dyp1* in Chinese cabbage.

### The Coding Sequence and Expression Pattern of *BraA07.ZEP* Show Only a Little Difference Between Dark Yellow and Yellow Petals

Given that the coding sequence of *BraA07.ZEP* (*Bra012127*) was very similar to that of *Br-dyp1* (*BraA09.ZEP*), with 87.02% identity, we designed a primer pair, ZEP-A07-ful-F and ZEP-A07-ful-R (Supplementary Table 8), to amplify the full-length CDS of *BraA07.ZEP* in R16-11 and SD369. The CDS of *BraA07.ZEP* from R16-11 and SD369 were submitted to GenBank under the accession numbers OL436222 (R16-11) and OL436223 (SD369). Sequence alignment revealed that there were 29 SNP variations between the coding sequences of R16-11 and SD369 (Supplementary Table 10; Supplementary Figure 5). Among the 29 SNPs, 27 SNPs were synonymous mutations, and only 2 SNPs caused non-synonymous mutations, which did not affect the protein function (Supplementary Table 10; Supplementary Figure 6). The expression pattern of *BraA07.ZEP* in the parental lines was also checked using primer pair ZEP-A07-qF and ZEP-A07-qR. The results revealed that the highest transcript levels were detected in the leaves, whereas low levels were found in petals and other tissues (Figure 4B). Furthermore, the expression of *BraA07.ZEP* in the petals of R16-11 was only slightly higher than that in SD369 (Figure 4B), which indicated a functional divergence between *BraA07.ZEP* and *Br-dyp1* (*BraA09.ZEP*).

### Transcriptome Analysis in Dark Yellow and Yellow Petals

To examine the global effect of the *Br-dyp1* (*BraA09.ZEP*) mutation on gene expression in Chinese cabbage, RNA-seq analysis was employed to profile gene expression differences in petals from the Y-bulk and DY-bulk, each with three replicates. Approximately 290 million clean reads were generated for the six samples and 83.9–87.5% were uniquely mapped to the *B. rapa* (Chiifu-401) reference genome (Supplementary Table 5). All the clean reads were deposited in the NCBI Short Read Archive database under accession number PRJNA779176. Statistical analysis identified 835 DEGs with at least two-fold changes between the Y-bulk and DY-bulk from the three biological replicates (FDR ≤ 0.05). Among these DEGs, 248 genes were upregulated and 587 genes were downregulated in the dark yellow petals.

**Figure 6:**
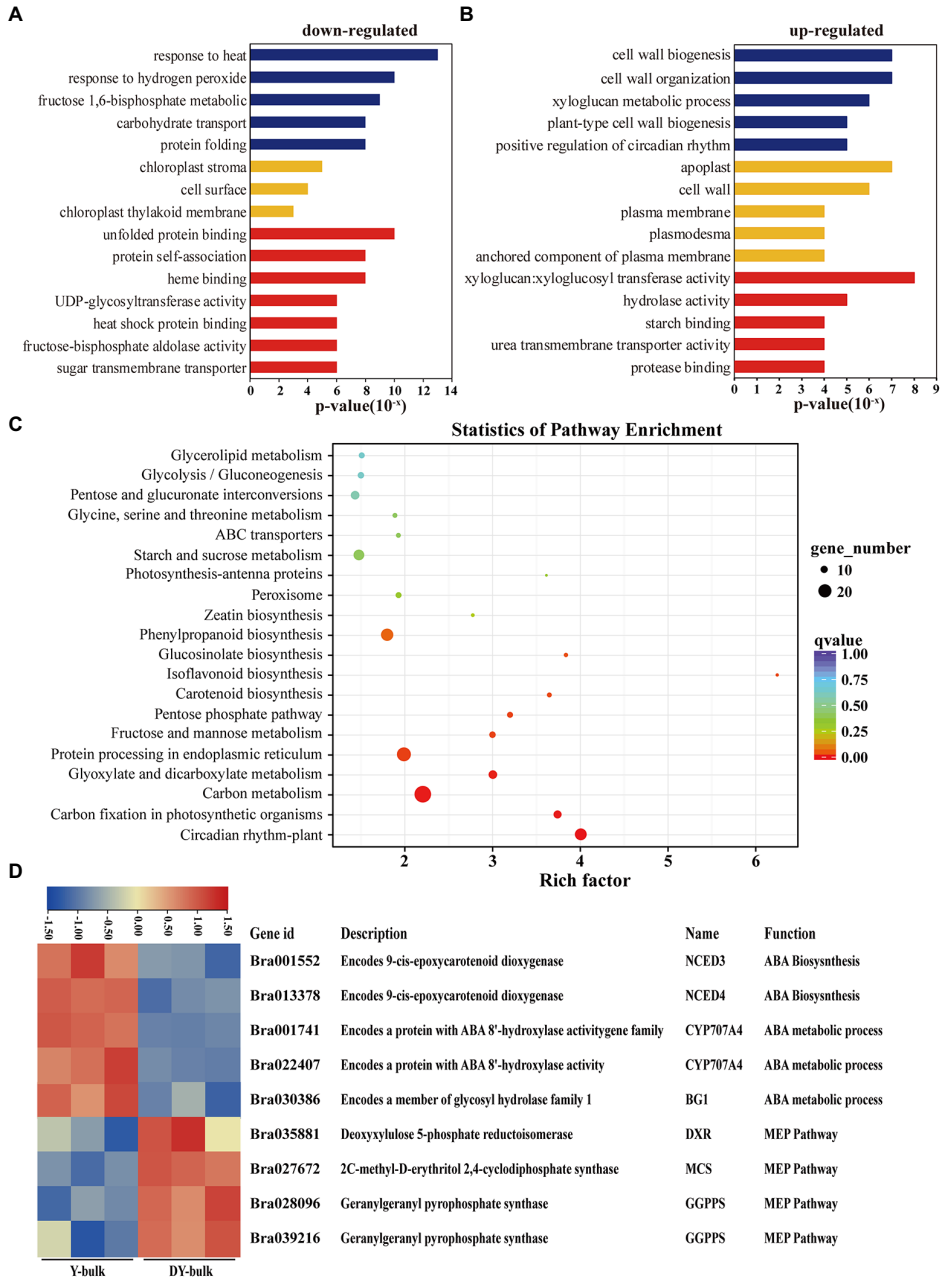
Transcriptome analysis in yellow and dark yellow petals. **(A,B)** GO terms that were significantly enriched in 587 downregulated genes **(A)** and in 278 upregulated genes in the DY-bulk **(B)**. **(C)** Scatter plot of top 20 enriched KEGG pathways. Rich factor is the ratio of the DEG number to the background number in a certain pathway. The size of the dots represents the number of genes, and the color of the dots represents the range of the *q*-value. **(D)** Differentially expressed genes related to the carotenoid metabolism and flux. The heatmap colors are shown in log2 (FPKM). Three biological replicates of the W-bulk and G-bulk are shown.

GO enrichment analysis of the 587 downregulated genes revealed that most DEGs were assigned to the “chloroplast stroma (GO: 0009570)” and “chloroplast thylakoid membrane (GO: 0009535)” terms in the cellular component category (Figure 6A), which was compatible with the ZEP localization in chloroplasts (Rock et al., 1992). Zeaxanthin is involved in non-photochemical quenching (NPQ) and thylakoid stacking, and thus affects the PSII function (Rock et al., 1992). Additionally, zeaxanthin serves important functions as an antioxidant in the lipid phase of the membrane and is likely to act as a key component in the memory of the chloroplast with respect to preceding photo-oxidative stress (Jahns and Holzwarth, 2012). In this study, genes participating in the “response to heat (GO: 0009408),” the “response to hydrogen peroxide (GO: 0042542),” the “fructose 1,6-bisphosphate metabolic process (GO: 0030388),” and “carbohydrate transport (GO: 0008643)” were significantly enriched in the biological process category (Figure 6A), which implied that due to the increased accumulation of zeaxanthin in dark yellow petals, the photo and heat stress and reactive oxygen were removed, so the genes involved in heat and hydrogen peroxide responses were downregulated. Furthermore, due to the sustained energy dissipation by zeaxanthin, genes with function of “UDP-glycosyltransferase activity (GO: 0008194)” and “sugar transmembrane transporter activity (GO: 0051119)” were downregulated (Figure 6A). Interestingly, GO enrichment analysis of the 248 upregulated genes showed that “cell wall biogenesis (GO: 0042546),” “cell wall organization (GO: 0071555), and “xyloglucan metabolic process (GO: 0010411)” were the top three significantly enriched terms in the biological process category, and large proportions of DEGs were assigned to the “apoplast (GO: 0048046)” and “cell wall (GO: 0005618)” in the cellular component category (Figure 6B). Xyloglucan endotransglucosylase/hydrolase genes (*XTHs*), which encode proteins with xyloglucan:xyloglucosyl transferase activity (GO: 0016762; Figure 6B), are involved in petal abscission in rose (Singh et al., 2011). In our study, the upregulated expression of *XTHs* in dark yellow petals might have caused faster petal abscission to save energy and to compensate sustained energy dissipation by zeaxanthin.

KEGG pathway enrichment analysis revealed that “carotenoid biosynthesis” was one of the most significantly enriched pathways (Figure 6C). When focusing on specific genes, in addition to the downregulation of the candidate gene *Bra037130* (*BraA09.ZEP*) in the DY-bulk, five genes involved in carotenoid degradation and ABA biosynthetic and metabolic processes, including *NECD3* (*Bra001552*), *NCED4* (*Bra013378*), *CYP707A4* (*Bra001741* and *Bra022407*) and *BG1* (*Bra030386*; Figure 6D), were also found to be significantly downregulated (FDR ≤ 0.05 and |log2 (fold change)| ≥ 1). Notably, most genes involved in carotenoid biosynthesis, such as *PSY* (*Bra006391*), *PDS* (*Bra010751*), *ZDS* (*Bra040411*), *LCYB* (*Bra022892* and *Bra029825*), and *LCYE* (*Bra006838*), were slightly downregulated (FDR ≤ 0.05 and |log2 (fold change)| < 1; Supplementary Table 11). However, some of the genes in the MEP pathway, such as *DXR* (*Bra035881*), *MCS* (*Bra027672*), and *GGPPS* (*Bra028096* and *Bra039216*; Supplementary Table 11), were slightly upregulated, which might partially contribute to the increased total carotenoid levels. In recent years, increasing evidence of cross-talk between flavonoid and carotenoid pathways has been obtained (Davuluri et al., 2005; Meng et al., 2019; Liu et al., 2020b). In this study, most genes involved in isoflavonoids and flavonoids biosynthesis were downregulated (Supplementary Table 11), which was consistent with the previous study (Liu et al., 2020b). The “circadian rhythm” pathway was significantly enriched, and most of the genes in this pathway were downregulated (Supplementary Table 11), which concurred that the transcription of ZEP gene follows a diurnal rhythm (Audran et al., 1998; Thompson et al., 2000). Furthermore, genes in the “carbon fixation in photosynthetic organisms” pathway were significantly enriched, and most of these genes were downregulated (Supplementary Table 11), which was consistent with the results of GO enrichment analysis, suggesting that the loss-of-function of ZEP caused increased accumulation of zeaxanthin and disturbed antenna assembly and affected the photosynthesis system.

## Discussion

The present study successfully fine-mapped the *Br-dyp1* gene to a physical interval of 53.6 kb. Many lines of evidence revealed that the *Bra037130* (*BraA09.ZEP*) gene is the candidate gene for *Br-dyp1*. First, the functional annotation analysis of 11 genes within the 53.6 kb interval revealed that only one gene, *Bra037130*, homologous to *ZEP* in *Arabidopsis*, was involved in carotenoid biosynthesis. Second, the expression level of *Bra037130* (*BraA09.ZEP*) in flower tissues was much lower in deep yellow petals than in yellow petals. Third, sequence alignment showed that the 679 bp insertion in dark yellow petals caused a premature stop codon, thus causing the loss of function of the ZEP enzyme. Most importantly, we developed a functional marker for the candidate gene, and validation showed that this functional marker co-segregated with the petal color phenotype. Furthermore, carotenoid profile analysis showed increased accumulation of zeaxanthin and a reduction of violaxanthin, which concurred with the ZEP function (Marin et al., 1996; Gonzalez-Jorge et al., 2016). Above all, a 679 bp insertion of *Bra037130* (*BraA09.ZEP*) in SD369 was the main reason that caused the dark yellow petal color phenotype. The developed functional marker can be used for molecular-assisted breeding and for developing new ornamental varieties with visual appeal, which has profound significance.

The gene structure annotation of *Bra037130* (*BraA09.ZEP*) in *B. rapa* V1.5 reference genome has some mistake. When we cloned the candidate gene *Bra037130* (*BraA09.ZEP*) for *Br-dyp1*, we first designed the primer pair Br-dyp1-F and Br-dyp1-R1 (Supplementary Table 8) to amplify the full-length sequence according to the *B. rapa* V1.5 reference genome. The results showed that no amplification products could be detected using cDNA from SD369 or R16-11 as a template. Another fragment-amplifying primer pair Br-dyp1-qF2 and Br-dyp1-qR2 (Supplementary Table 8), which targeted the eighteenth exon of *Bra037130* according to the *B. rapa* V1.5 annotation, were designed and still no amplification products were detected using cDNA from either parent as the templates. However, the qRT-PCR results (Figure 4A) and the RNA-seq analysis (Figure 5D) showed that the candidate gene *Bra037130* did express in the parental lines. Thus, there must be some mistake in the *Bra037130* annotation in *B. rapa* V1.5. We subjected the CDS of *Bra037130* to a BLAST search against the *B. rapa* V3.0 reference genome and found that the gene corresponding to *Bra037130* in *B. rapa* V3.0 was *BraA09g009220.3C*. The *BraA09g009220.3C* gene owned 14 exons and 13 introns, whereas *Bra037130* had 18 exons and 17 introns (Supplementary Table 12), indicating a considerable difference between the *B. rapa* V1.5 and V3.0 annotations. Therefore, we designed another full-length primer pair BrZEP-ful-F and BrZEP-ful-R2 to amplify the full-length sequence according to the *B. rapa* V3.0 reference genome, and both the full-length gDNA and cDNA could be amplified in the two parent lines (Supplementary Figure 2). Thus, the gene structure annotation of *Bra037130* in *B. rapa* V3.0 reference genome is corrected according to our experiments results.

Through LC-APCI-MS/MS analysis with saponification, we observed that lutein was the most abundant carotenoid in yellow petals, whereas violaxanthin was the second most abundant carotenoid, accounting for approximately 57.7 and 24.1% of the total carotenoids in the Y-bulk, respectively. These results differed from our previous study (Yang et al., 2021) and another study conducted in Chinese cabbage (Zhang et al., 2020), in which the violaxanthin was the most abundant carotenoid and lutein was the second most abundant carotenoid. It has been reported that deepoxidation to zeaxanthin is favored in high-light conditions, while epoxidation to violaxanthin predominates under moderate-light conditions (Kalituho et al., 2007; Gonzalez-Jorge et al., 2016). The reasons for the above difference might due to the different seasons and different places for the petal sample collection. Because petals were collected in November and in greenhouse in our previous study (Yang et al., 2021), in which the sunlight was mild and the epoxidation to violaxanthin was favored, whereas we collected the petals in June and in an open field for this study, where the sunlight was bright and strong, so the de-epoxidation to zeaxanthin dominated.

A common feature of *zep* mutant leaves is the increased accumulation of zeaxanthin and decreased production of antheraxanthin and violaxanthin (Rock and Zeevaart, 1991; Marin et al., 1996; Niyogi et al., 1998; Liu et al., 2020b). As expected, in our study, the amount of zeaxanthin increased about 11.2-fold in DY-bulk than that in Y-bulk, and violaxanthin decreased about 3.6-fold in DY-bulk. Unexpectedly, the antheraxanthin increased about 11.5-fold in DY-bulk, which was different from the findings of previous studies (Rock and Zeevaart, 1991; Marin et al., 1996; Gonzalez-Jorge et al., 2016). We speculated that there were at least two mechanisms that might explain this difference. First, the enzyme zeaxanthin epoxidase might show substrate specificity. *Phaeodactylum tricornutum* contains three copies of *ZEP*, which exhibit different catalytic activities and substrate specificities (Eilers et al., 2016). In this study, the mutation of *Br-dyp1* (*BraA09.ZEP*) mainly disturbed the epoxidation from antheraxanthin to violaxanthin, and the enzyme encoded by *Br-dyp1* (*BraA09.ZEP*) might therefore exhibit substrate specificity for antheraxanthin. Second, the genome of Chinese cabbage has undergone genome triplication, and another paralogous gene, *BraA07.ZEP*, might contribute to epoxidation from zeaxanthin to antheraxanthin. Zeaxanthin epoxidases is present in only one gene copy in the model plants *Arabidopsis* or rice (Rock and Zeevaart, 1991; Agrawal et al., 2001). However, there were two copies of *ZEP* found in Chinese cabbage, and the sequence identity between *BraA07.ZEP* and our candidate gene, *Br-dyp1* (*BraA09.ZEP*), was high to 87.02% in CDS. *BraA07.ZEP* was mainly expressed in leaves, whereas *Br-dyp1* (*BraA09.ZEP*) was mainly expressed in flower tissues, the tissue-specific expression pattern showed a functional divergence, which was consistent with the results obtained in *B. napus* (Liu et al., 2020b). Although *BraA07.ZEP* was mainly expressed in leaves, its transcripts could also be detected in petals. Hence, we suspected that the enzyme encoded by *BraA07.ZEP* might partially compensate for the loss-of-function mutation of *Br-dyp1* (*BraA09.ZEP*) in dark yellow petals and might be responsible for epoxidation from zeaxanthin to antheraxanthin. A transgenic line of *A. thaliana* with partly disabled zeaxanthin epoxidase activity also showed increased levels of zeaxanthin and antheraxanthin and decreased levels of violaxanthin (Nowicka et al., 2009), which is same with our results, and further confirming our above speculation.

In addition to the increased zeaxanthin and antheraxanthin, other xanthophylls especially the lutein increased about 6.7-fold in DY-bulk, and the total content of carotenoids showed a remarkable 4.7-fold increase in the DY-bulk, which can also be observed in maturing *Arabidopsis* seeds (Gonzalez-Jorge et al., 2016). Two possible reasons might explain this result. First, the enzyme ZEP is not only responsible for the epoxidation from zeaxanthin to violaxanthin, but may also be responsible for the epoxidation from lutein to lutein epoxide. Lutein epoxide is widespread among photosynthetic and non-photosynthetic plant tissues (Garcia-Plazaola et al., 2007), which has been detected in chromoplasts from flowers (Tai and Chen, 2000), fruits (Watanabe and Takahashi, 1999), seeds (Edelenbos et al., 2001), and tubers (Lu et al., 2001). The loss of ZEP activity in dark yellow petals disturbed not only the xanthophyll cycle but also the lutein epoxide cycle, thus causing increases in the accumulation of zeaxanthin and lutein. Second, the mutation of the gene *Br-dyp1* (*BraA09.ZEP*) blocked the carotenoid flux, impaired the carotenoid degradation, and disturbed the flux from carotenoid to ABA biosynthesis, thus causing the bias from the β-branch to the α-branch, so the lutein increased and the total carotenoid increased. Our results were consistent with another study on a *ZEP* mutant, in which the lutein content was increased 2.2-fold and the total seed carotenoids showed a remarkable 6-fold increase relative to the wild type (Gonzalez-Jorge et al., 2016). In *B. napus*, the disruption of the gene *CCD4* impairs the carotenoid degradation and disturbs the carotenoid flux and causes the total carotenoid to increase approximately 42-fold, ultimately changing the flower color from white to yellow (Zhang et al., 2015). Finally, some genes in the MEP pathways were upregulated in the DY-bulk, while some genes for ABA biosynthesis were downregulated, which provided more precursors for carotenoid biosynthesis and alleviated the carotenoid degradation and loss, which might also contribute to the increased total carotenoids in dark yellow petals.

## Conclusion

The present study delimited the *Br-dyp1* gene responsible for the dark yellow petal color trait in Chinese cabbage. The *Br-dyp1* gene was fine-mapped to an interval of 53.6 kb *via* BSR-Seq and linkage analysis. Through functional annotation, expression profile and sequence variation analysis, *Bra037130* (*BraA09.ZEP*) which encodes a zeaxanthin epoxidase, was the most likely candidate gene for *Br-dyp1*. *BraA09.ZEP* is involved in the epoxidation from zeaxanthin to violaxanthin. A 679 bp insertion in dark yellow petals caused a premature stop codon, and, thus, the loss of ZEP enzyme function, which affected carotenoid metabolism and caused an increase in the accumulation of total carotenoids. Moreover, we developed and validated the functional marker Br-dyp1-InDel for *Br-dyp1*. This achievement is an important advance for molecular research on flower pigmentation in Chinese cabbage.

## Data Availability Statement

The datasets presented in this study can be found in online repositories. The names of the repository/repositories and accession number(s) can be found in the article/Supplementary Material.

## Author Contributions

X-WZ and YY conceptualized the experiments and provided the funding resource. SY drafted the manuscript. HL and YZ performed the experiments and analyzed the data. XW, HS, ZW, and XZ participated in drafting the article and revising it critically. All authors contributed to the article and approved the submitted version.

## Funding

This work was financially supported by Zhongyuan Scholar Program (202101510003), the China Agriculture Research System (CARS-23-G-15), Sci-Tech Innovation Team of Henan Academy of Agricultural Sciences (2021TD06), and the Self-dependent Innovation Program in Henan Academy of Agricultural Science (2121ZC23).

## Conflict of Interest

The authors declare that the research was conducted in the absence of any commercial or financial relationships that could be construed as a potential conflict of interest.

## Publisher’s Note

All claims expressed in this article are solely those of the authors and do not necessarily represent those of their affiliated organizations, or those of the publisher, the editors and the reviewers. Any product that may be evaluated in this article, or claim that may be made by its manufacturer, is not guaranteed or endorsed by the publisher.

## Supplementary Material

The Supplementary Material for this article can be found online at: https://www.frontiersin.org/articles/10.3389/fpls.2022.841328/full#supplementary-material

## Footnotes

^1^ http://www.metware.cn/
