# Supplementary material for: Map-Based Cloning and Characterization of Br-dyp1, a Gene Conferring Dark Yellow Petal Color Trait in Chinese Cabbage (Brassica rapa L. ssp. pekinensis)
Source: Front Plant Sci. 2022 Feb 17;13:841328. doi: 10.3389/fpls.2022.841328 (PMC8891484; doi:10.3389/fpls.2022.841328)
Supplement: Supplementary file 2 [file Presentation_1.PPTX]

## Slide 1
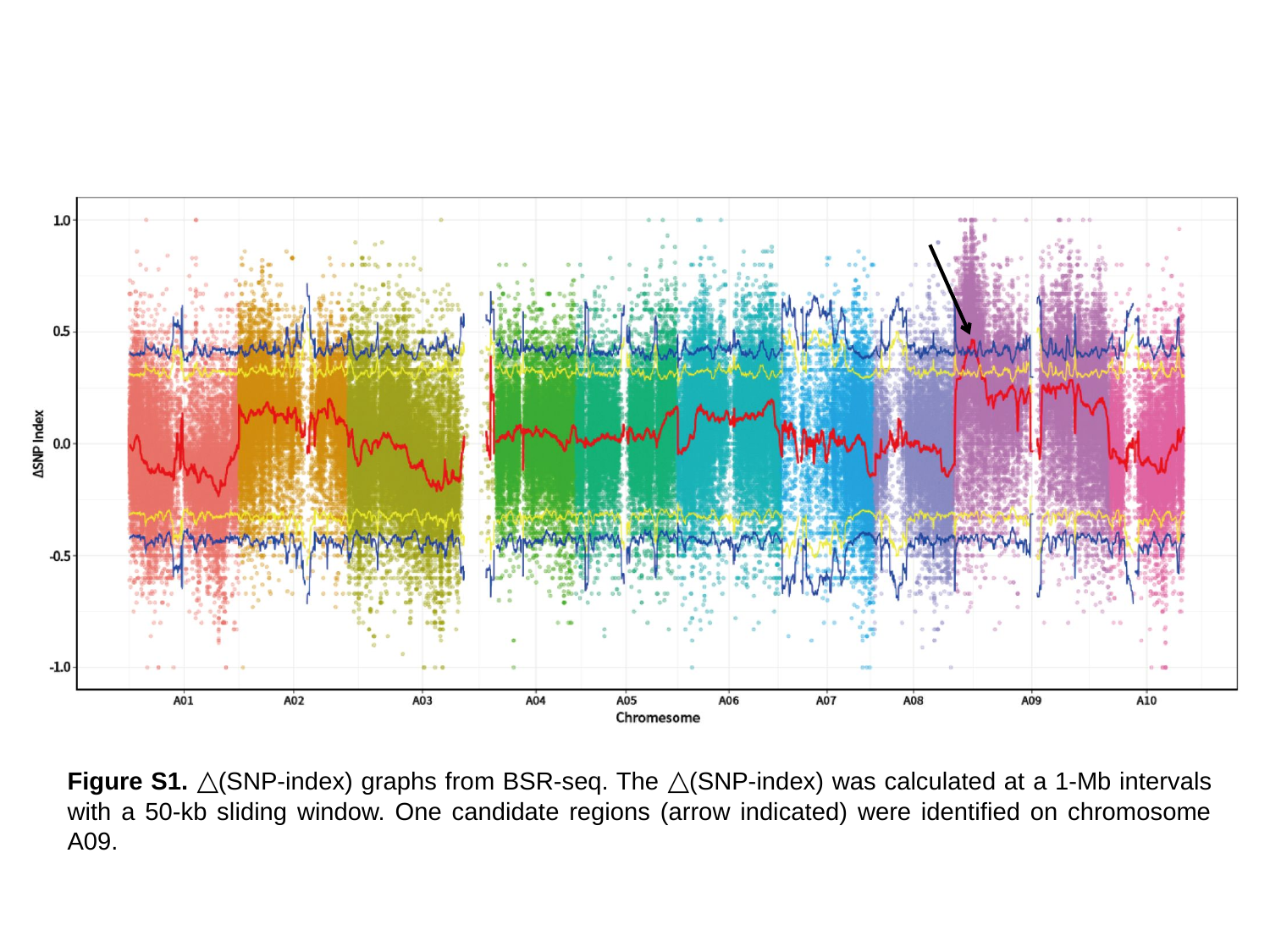

Figure S1. △(SNP-index) graphs from BSR-seq. The △(SNP-index) was calculated at a 1-Mb intervals with a 50-kb sliding window. One candidate regions (arrow indicated) were identified on chromosome A09.

## Slide 2
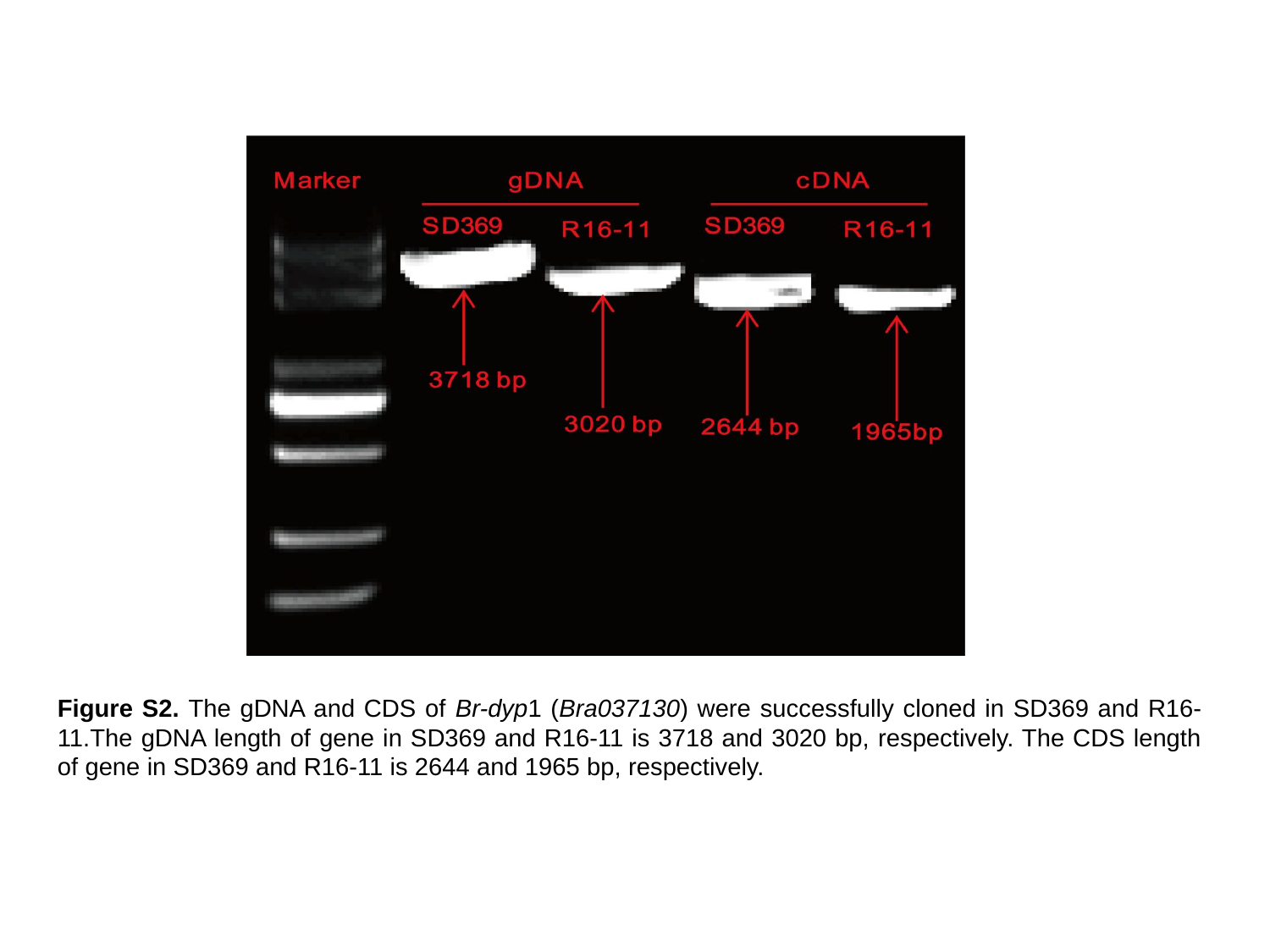

Figure S2. The gDNA and CDS of Br-dyp1 (Bra037130) were successfully cloned in SD369 and R16-11.The gDNA length of gene in SD369 and R16-11 is 3718 and 3020 bp, respectively. The CDS length of gene in SD369 and R16-11 is 2644 and 1965 bp, respectively.

## Slide 3
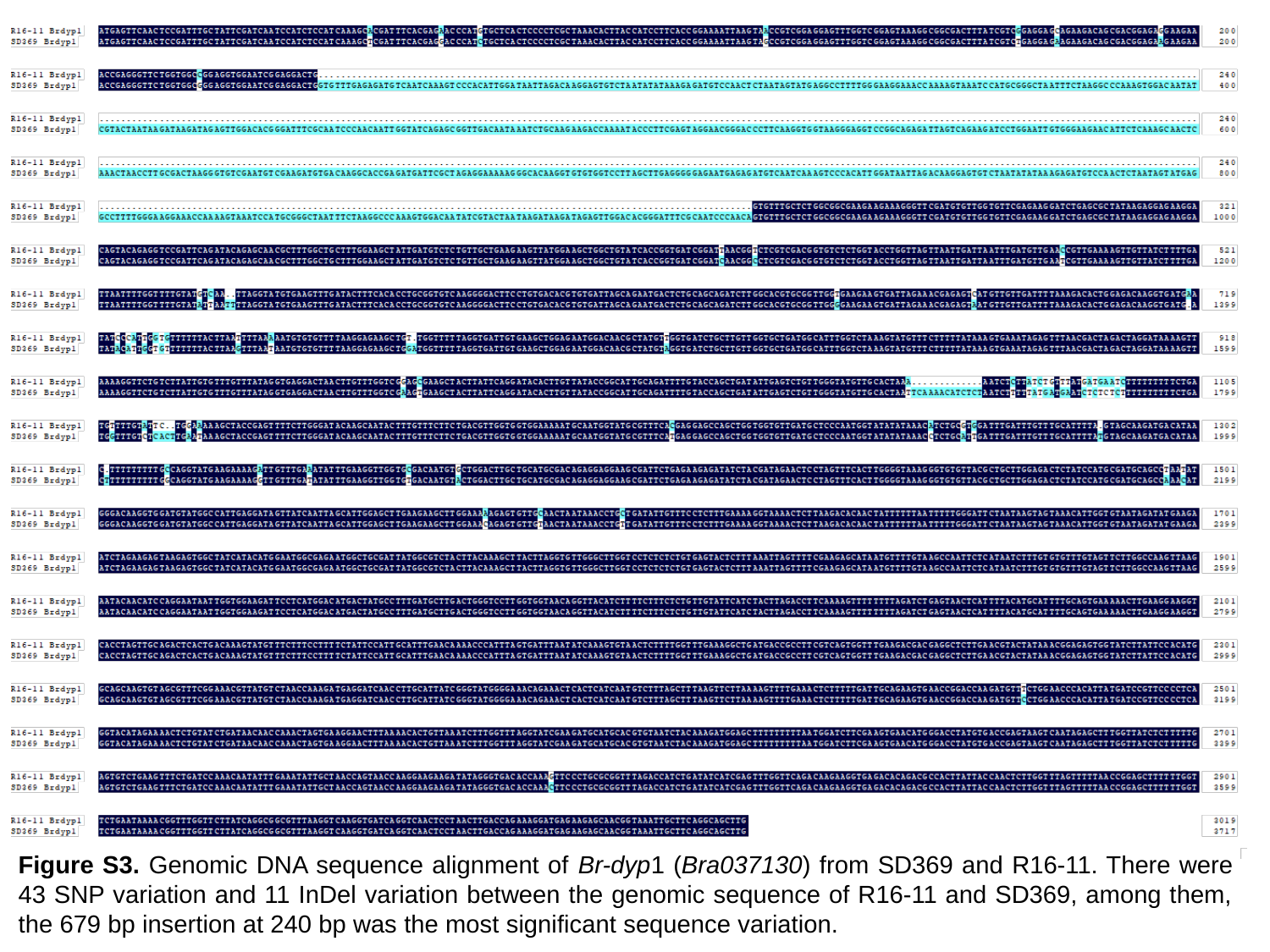

Figure S3. Genomic DNA sequence alignment of Br-dyp1 (Bra037130) from SD369 and R16-11. There were 43 SNP variation and 11 InDel variation between the genomic sequence of R16-11 and SD369, among them, the 679 bp insertion at 240 bp was the most significant sequence variation.

## Slide 4
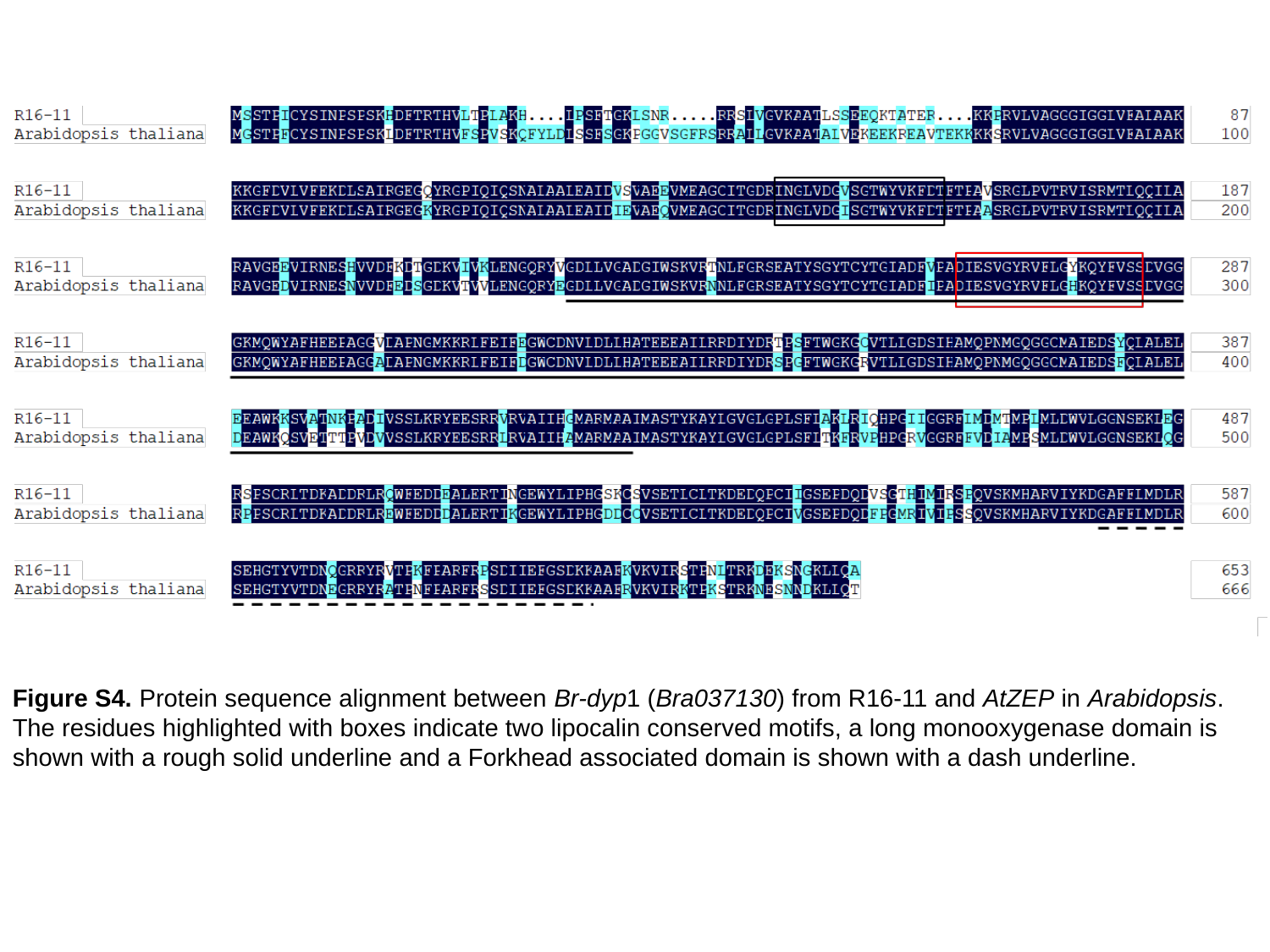

Figure S4. Protein sequence alignment between Br-dyp1 (Bra037130) from R16-11 and AtZEP in Arabidopsis. The residues highlighted with boxes indicate two lipocalin conserved motifs, a long monooxygenase domain is shown with a rough solid underline and a Forkhead associated domain is shown with a dash underline.

## Slide 5
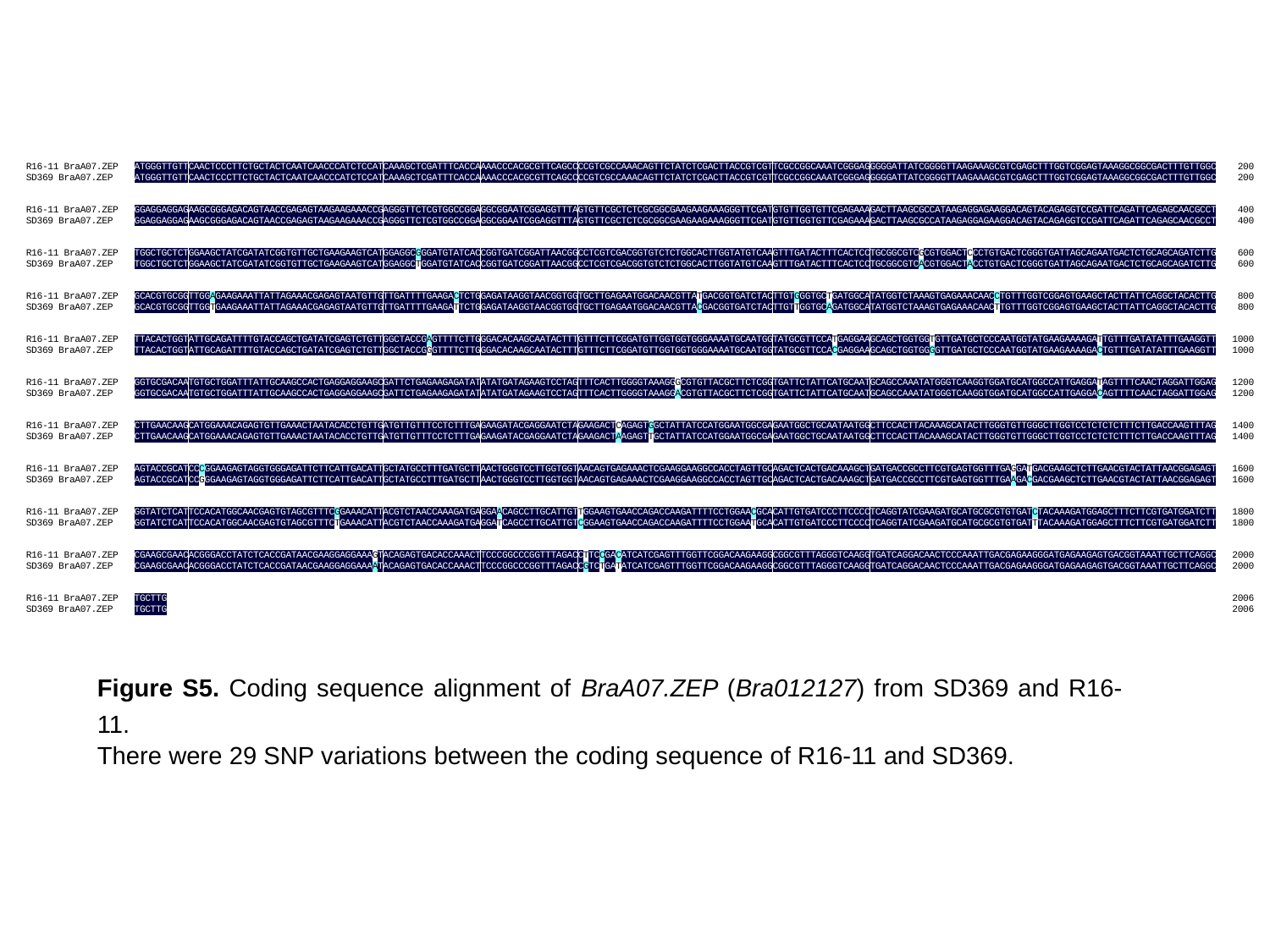

Figure S5. Coding sequence alignment of BraA07.ZEP (Bra012127) from SD369 and R16-11.
There were 29 SNP variations between the coding sequence of R16-11 and SD369.

## Slide 6
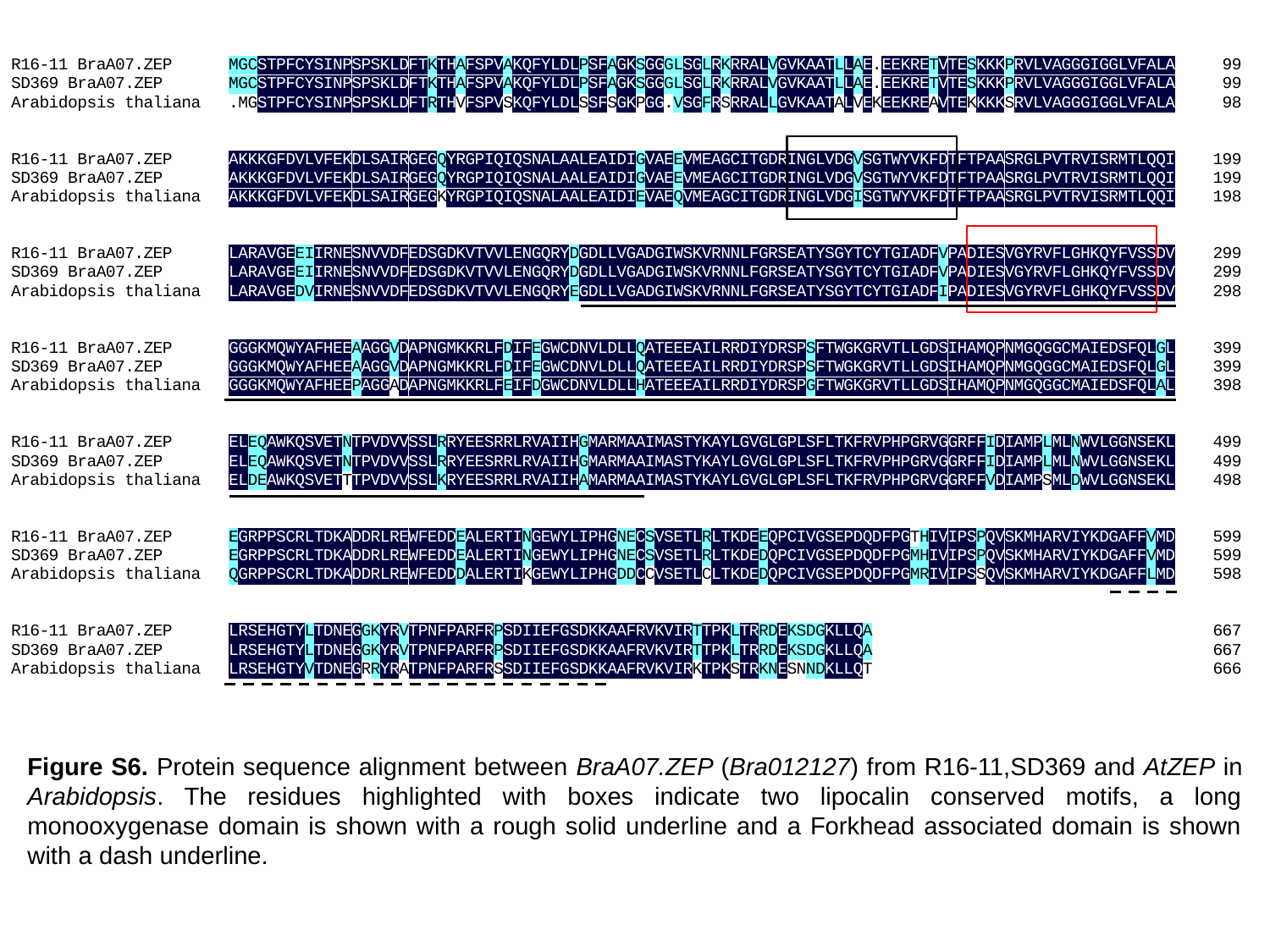

Figure S6. Protein sequence alignment between BraA07.ZEP (Bra012127) from R16-11,SD369 and AtZEP in Arabidopsis. The residues highlighted with boxes indicate two lipocalin conserved motifs, a long monooxygenase domain is shown with a rough solid underline and a Forkhead associated domain is shown with a dash underline.
